# FIBOS: R and python packages for analyzing protein packing and structure

**DOI:** 10.1093/bioinformatics/btaf434

**Published:** 2025-08-04

**Authors:** Herson H M Soares, João P R Romanelli, Patrick J Fleming, Carlos H da Silveira

**Affiliations:** Institute of Technological Sciences, Federal University of Itajubá, Itabira, 35903-087, Brazil; Institute of Applied and Pure Sciences, Federal University of Itajubá, Itabira, 35903-087, Brazil; Thomas C. Jenkins Department of Biophysics, Johns Hopkins University, Baltimore, MD 21218, United States; Institute of Technological Sciences, Federal University of Itajubá, Itabira, 35903-087, Brazil

## Abstract

**Motivation:**

Advances in the prediction of the 3D structures of most known proteins through machine learning have achieved unprecedented accuracies. However, although these computed models are remarkably good, they still challenge accuracy at the atomic level. The Occluded Surface (OS) algorithm is widely used for atomic packing analysis. But it lacks implementations in high-level languages.

**Results:**

We introduce FIBOS, an R and Python package incorporating the OS methodology with enhancements. We show how FIBOS can be used to atomically compare experimental structures and AlphaFold predictions. Although the average packing was similar, AlphaFold models exhibited slightly greater variability, revealing a specific pattern of outliers.

**Availability and implementation:**

FIBOS can be installed locally as a PyPi Python or CRAN R package, and it is also available at https://github.com/insilico-unifei/fibos-R and https://github.com/insilico-unifei/fibos-py.

## 1 Introduction

Advances in protein structure prediction by machine learning methods have made estimating the 3D shape of virtually all known protein sequences a reality, with unprecedented accuracy (Jänes and Beltrao 2024). However, these advances introduce new challenges in low-level accuracy, especially the fine-tuning of the atomic positions (Binbay *et al.* 2023).

Molecule packing density calculations, in particular, play a crucial role in the structural analysis and assessment of protein models (Fleming and Richards 2000). Accurate packing density reflects the efficient organization of atoms, correlating directly with structural stability, functional integrity, and the realistic representation of interactions. Discrepancies in packing density can indicate potential inaccuracies, such as misaligned side chains, unrealistic voids, or incorrect folding, which can compromise the functional predictions of the model (Sonavane and Chakrabarti 2008). They are also important for algorithms that depend on plausible atomic coordinates for building reliable contact networks (da Silveira *et al.* 2009).

**Figure 1.**
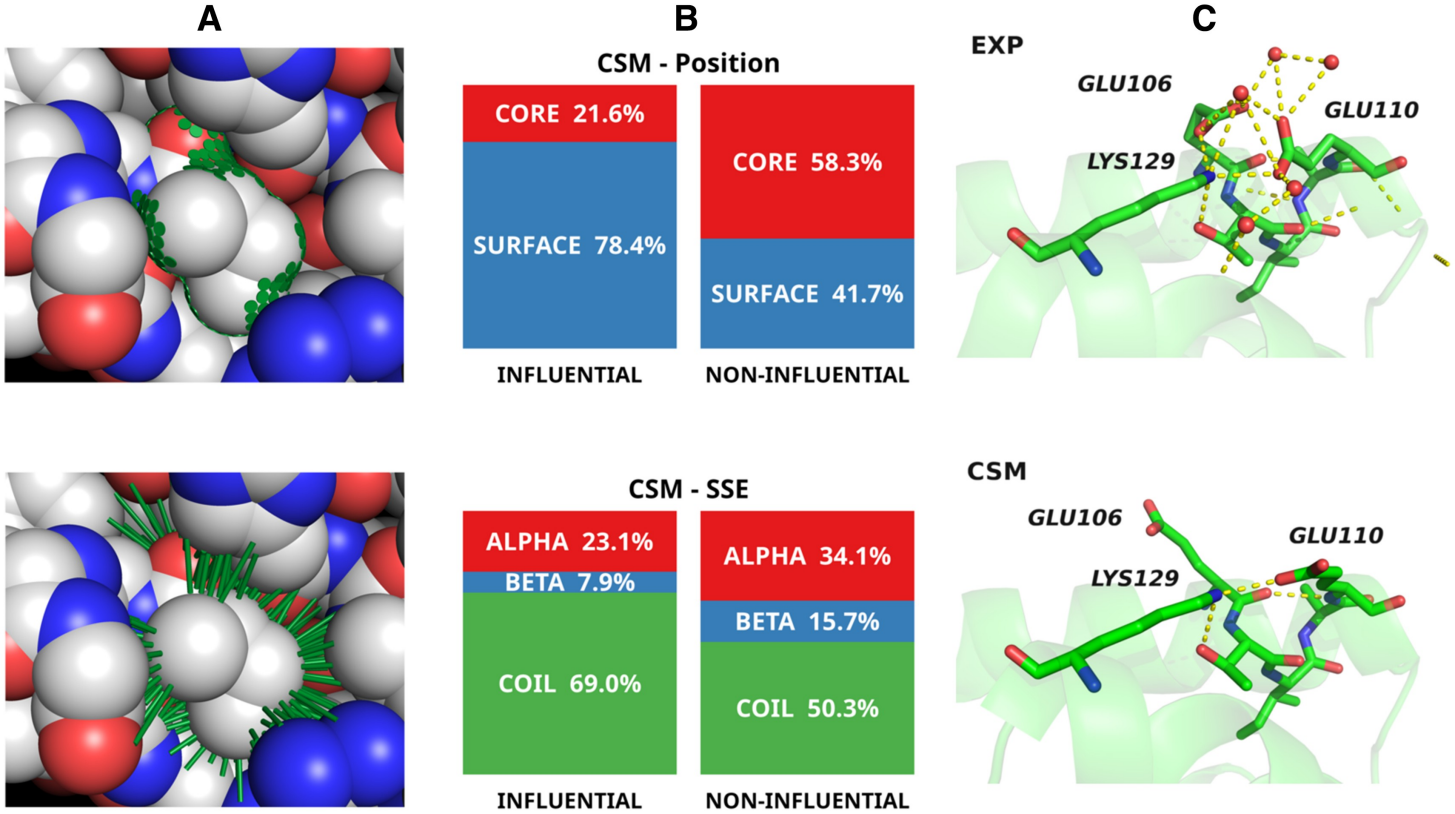
Illustration of the OS method and comparison of computed structural models versus experimentally determined protein models. (A) (upper panel), OS dots; (A) (lower panel), OS normals for ILE44 of ubiquitin 1UBQ; (B), the influential residues that contribute most to the increased standard deviation of OSP in computed structural models are predominantly surface exposed (upper panel) and found in coil regions of the protein (lower panel); (C), the surface residue GLU110 in the experimentally determined structure of 1NG6 is part of a cluster of charges, with GLU106, GLU110, LYS129, and waters forming a network of hydrogen bonds (upper panel); in contrast, in the computed structural model there are no mediating waters contributing to an attractive/repulsive imbalance between the charged residues. As a consequence, GLU106 extends away from GLU110 (lower panel). Structural images made with Pymol (DeLano 2015).

To date, one of the best-known algorithms for atomic packing analysis is Occluded Surface (OS) (Pattabiraman *et al.* 1995). This method distributes dots (representing patches of area) across the atom surfaces. Each dot has a normal that extends until it reaches either a van der Waals surface of a neighboring atom (the dot is considered occluded) or covers a distance greater than the diameter of a water molecule (the dot is considered non-occluded and disregarded). As a consequence, buried and well-packed atoms, such as those in the protein core or in hot spot regions at chain-chain interfaces, tend to accumulate more dots on their surface and shorter normal lengths (Fig. 1A). On the other hand, those that are less packed, like near cavities or unstructured regions, may have fewer dots and longer normals. Thus, with the summed areas of dots and the lengths of normals, it is possible to compose robust metrics capable of inferring the average packing density of atoms, residues, and proteins, as well as any other group of biomolecules.

However, there are still no OS implementations in higher-level languages, such as R and Python. Both are fast and efficient coding languages with a wide user base across common operating systems and are especially suited to handling large volumes of data and analysis. Given that hundreds of millions of protein structure predictions are being generated by machine learning systems (Callaway 2022), having atomic packing density checking algorithms like OS in R and Python is a useful addition to available analytical tools.

Here, we present FIBOS, a package for biomolecule packing estimation in R and Python. It has embedded the same efficient Fortran implementations from the original OS but extended with some improvements. There are built-in functions that return useful data in tables, like dots and normals by atom or residue. Also, a function that implements the occluded surface packing density metric (OSP) averaged by residue, as described in (Fleming and Richards 2000). Another important advancement concerns the algorithm for generating dots onto the surface of an atom. The original OS covered the surface radially with one of the axes as a reference, introducing not only axial anisotropies but also inhomogeneities in the dot areas when comparing poles and equator. In this version, Fibonacci spirals were used to allocate the dots, which is known to produce lower axial anisotropy as well as more evenly spaced points on a sphere (Swinbank and Purser 1999). Some comparative analysis between OS and FIBOS can be seen in Fig. 1A to 2B, available as supplementary data at *Bioinformatics* online. The user can choose between classical OS or new FIBOS (default) methodologies. The FIBOS package is multi-platform and runs on Linux, Windows and Mac.

## 2 Methods

See Supplementary Information (SI), available as supplementary data at *Bioinformatics* online.

## 3 Results

To validate the libraries in R and Python, the average OSP of the validation dataset mentioned in SI was calculated and the results compared with pure Fortran. Fig. 3, available as supplementary data at *Bioinformatics* online presents linear regressions using these 3 programming languages, revealing a high correlation above 0.99. This attests to the reliability of the values calculated in R and Python.

To evaluate the potential use of the new libraries in R and Python, a case study was set up, aiming to compare the packing density between experimentally determined structures (EXP) and the equivalent computed structure models (CSM) predicted by AlphaFold (AF) (Varadi *et al*. 2022). To this end, a strict dataset with 261 chains was carefully assembled, with complete atomic and residual pairing and minimal bias related to size differences between CSM and EXP models (see SI, available as supplementary data at *Bioinformatics* online).

As can be seen in Fig. 6A, available as supplementary data at *Bioinformatics* online, the average OSP values are similar when considering CSM and EXP, given the analogous profile of central tendencies and distributions, with a high *P*-value in the Wilcoxon paired test (0.96) and a small Cohen’s d effect size (-0.02). The same is not true for the standard deviations (Fig. 6B, available as supplementary data at *Bioinformatics* online), revealing dissimilar points of central tendency and distributions, with a low Wilcoxon paired *P*-value (<0.001) and a near-medium Cohen’s d effect size (0.42). This indicates that the predicted CSM models, when compared with the experimental ones, may exhibit slightly greater variability and less homogeneity, potentially reflecting local divergences.

In order to better characterize such dissimilarities, we used influence functions (Hampel 1974) to identify the residues with the greatest contributions to the differences in standard deviations (SD) between the models (see SI, available as supplementary data at *Bioinformatics* online). Approximately 2% (on median), but up to 18% of the residues were considered influential, those that contributed the most to the greater dispersion of CSM versus EXP (see Fig. 10B, available as supplementary data at *Bioinformatics* online). From Fig. 1B, it can be seen that such residues predominated on the surface and in unstructured regions. More details on a comparison of influential and non-influential residues are illustrated in Figs. 6 and 7 and Tables 2 to 5, available as supplementary data at *Bioinformatics* online.

A real-world case illustrating differences between CSM and EXP structures can be seen in Fig. 1C. The surface residue GLU110 is the one with the greatest influence on the SD differences in 1NG6. In the EXP structure, GLU110 composes a cluster of charges, with GLU106, LYS129, and waters forming a network of hydrogen bonds. In the CSM structure, there are no mediating waters, contributing to an attractive/repulsive imbalance between the charged residues. As a consequence, GLU106 disaggregates from the cluster.

Another example involves the buried influent LEU16 of 1PZ4. In the CSM model (unlike the EXP), the approach of a PHE to LEU16 partially obstructs the entrance to the catalytic pocket (Fig. 9, available as supplementary data at *Bioinformatics* online). Since all CSM models are in apo form, the formation of the PHE/LEU pair within the pocket may be a consequence of conformational flexibility (Dyer *et al.* 2003). But, if the CSM structure were the only one available, this partially obstructed pocket could be a complication for docking or functional inference algorithms.

These examples show how FIBOS can be used to capture subtle but important conformational differences between CSM and EXP models.

## 4 Conclusion

We present here the FIBOS package for R and Python, capable of calculating the atomic packing density in proteins. The ease and speed of coding that such high-level languages provide is fundamental for the agility of analyses by researchers in structural biology. FIBOS can offer a reliable way to probe relevant structural divergences in a large number of biomolecules.

We show how FIBOS can be used to analyze differences between CSM and EXP models. Considering the atomic packing, the AF models offer quite satisfactory structural solutions. In general, the most influential residues involved in the EXP/AF variations are on the surface and in unstructured regions. In certain cases, these variations seem to be mainly due to the absence of water and ligands in the AF models. Overall, although AF offers good templates for initial structural studies, one should be cautious in using it without the appropriate conformational adjustments of residue side chains and explicit solvation.

## Supplementary Material

btaf434_Supplementary_Data

## Data Availability

Supplementary information is available at Bioinformatics online. Codes used in case study: https://github.com/insilico-unifei/fibos-R-case-study-supp and dataset also in: https://doi.org/10.5281/zenodo.15565375.
