# Supplementary material for: FIBOS: R and python packages for analyzing protein packing and structure
Source: Bioinformatics. 2025 Aug 4;41(9):btaf434. doi: 10.1093/bioinformatics/btaf434 (PMC12449057; doi:10.1093/bioinformatics/btaf434)
Supplement: btaf434_Supplementary_Data [file btaf434_supplementary_data.pdf]

# SUPPLEMENTARY INFORMATION

# FIBOS: R and Python packages for analyzing protein packing and structure

Herson H. M. Soares[1*], João P. R. Romanelli[2], Patrick J. Fleming[3], Carlos H. da Silveira[1*]

[1] Institute of Technological Sciences, Federal University of Itajubá,Campus Itabira, 35903-087, Brazil.

[2] Institute of Applied and Pure Sciences, Federal University of Itajubá, Campus Itabira, 35903-087, Brazil.

[3] Thomas C. Jenkins Department of Biophysics, Johns Hopkins University, Baltimore, MD, 21218, USA.

*Corresponding author: hersinsoares@gmail.com, carlos.silveira@unifei.edu.br.

## 1. VORONOI TILING

The differences of tiling on spheres between the classic OS and the new FIBOS are shown in Figs. S1A and S1B.

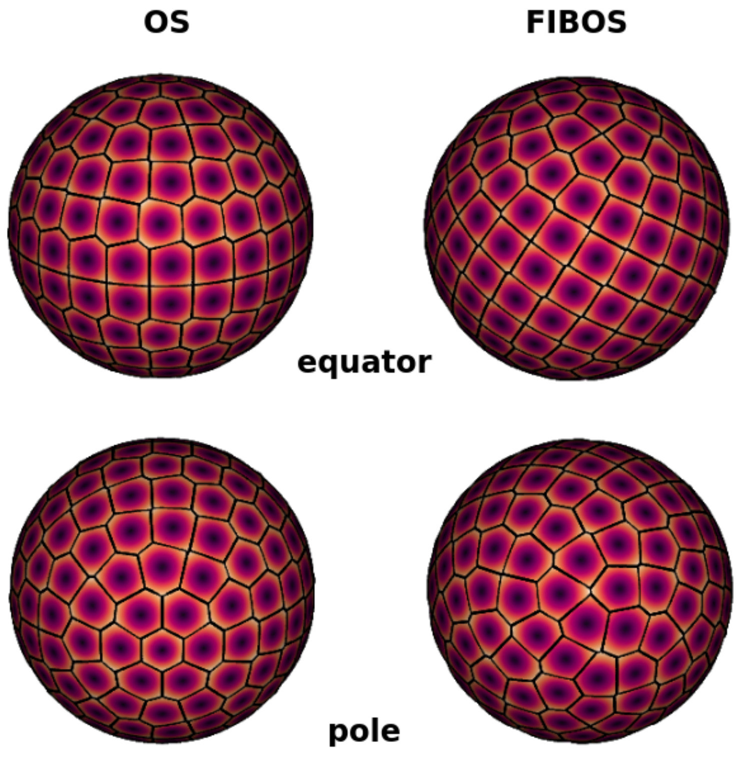


**Figure S1A.** Voronoi tiling on a sphere, comparing dot distributions for OS (left) and FIBOS (right). Above, it shows the equators; below, the poles. The spheres have a radius of 1.90 Å (equivalent to the van der Waals radius of carbon) and 212 dots were distributed, the default number used in the original OS.

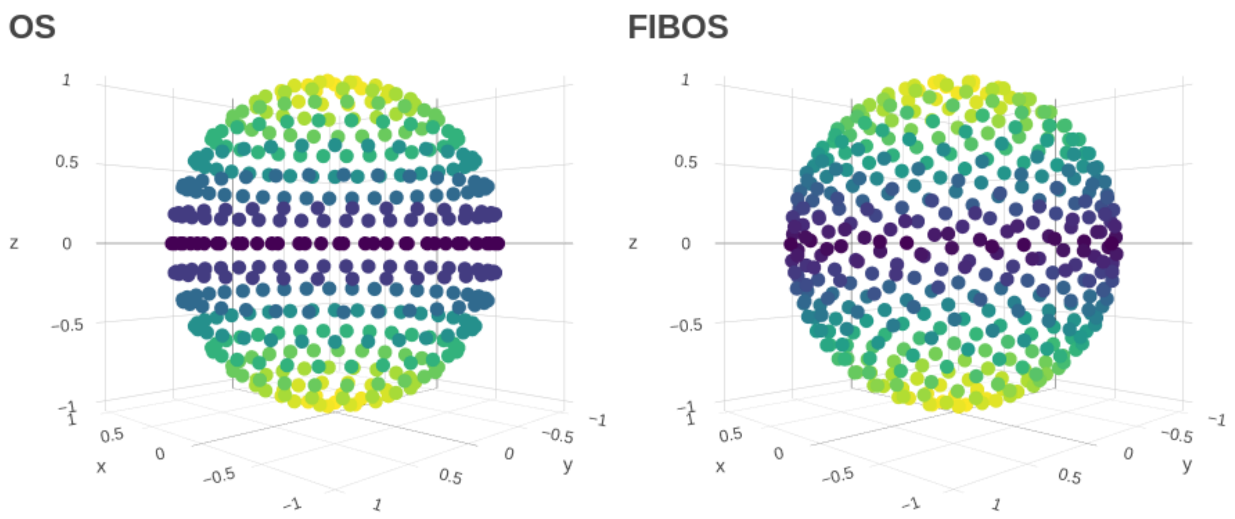


**Figure S1B.** Another way to see the Voronoi tiling on a sphere, for OS (left) and FIBOS (right). It is possible to see the anisotropy and bias in the distribution of points, more prominent in OS than FIBOS.

## 2. TILING AREA AND DOT DISTRIBUTION

The different Voronoi cell area and spherical cap distance distributions for FIBOS and OS are shown in Fig. S2A and S2B. FIBOS presents a profile of cell areas more concentrated around the mean value, indicating more evenly spaced points on a sphere. There is a bias in the distance distribution considering the spherical cap along the z axis, but it is much smaller in FIBOS than OS. FIBOS presents lower axial anisotropy than OS, especially for moderate numbers of dots.

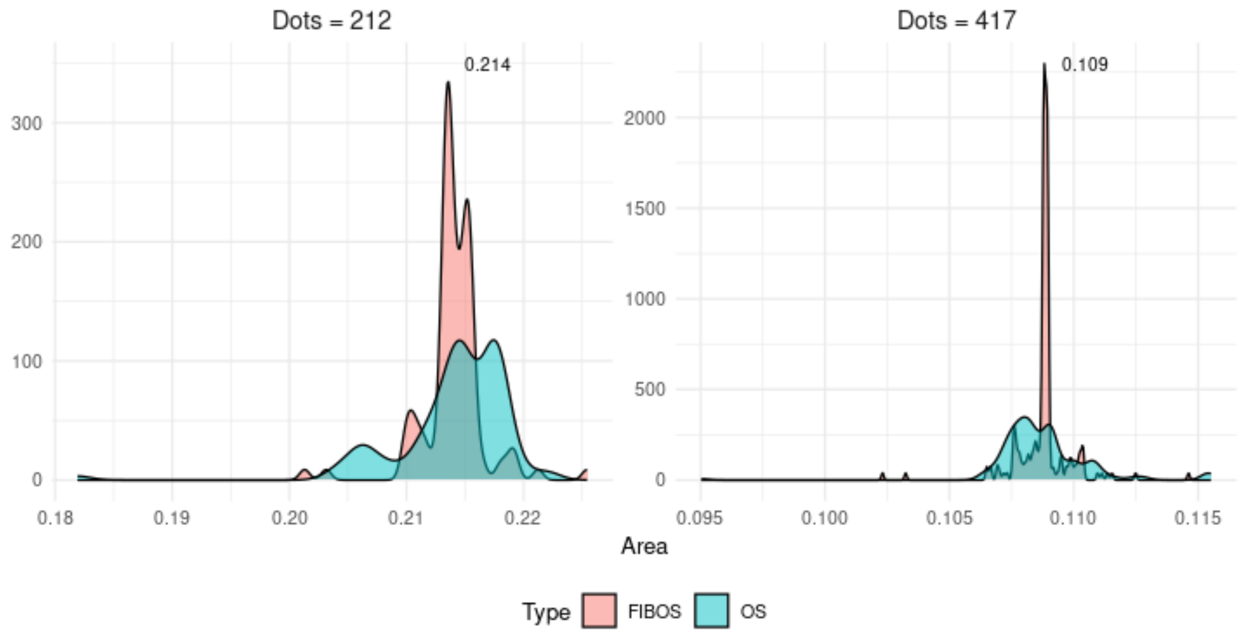


**Figure S2A.** Voronoi cell area distributions, comparing FIBOS and OS with 212 and 417 dots on a spherical surface of radius 1.90 Å (equivalent to the van der Waals radius of carbon). The numbers near to the modals depict the mean of cell areas.

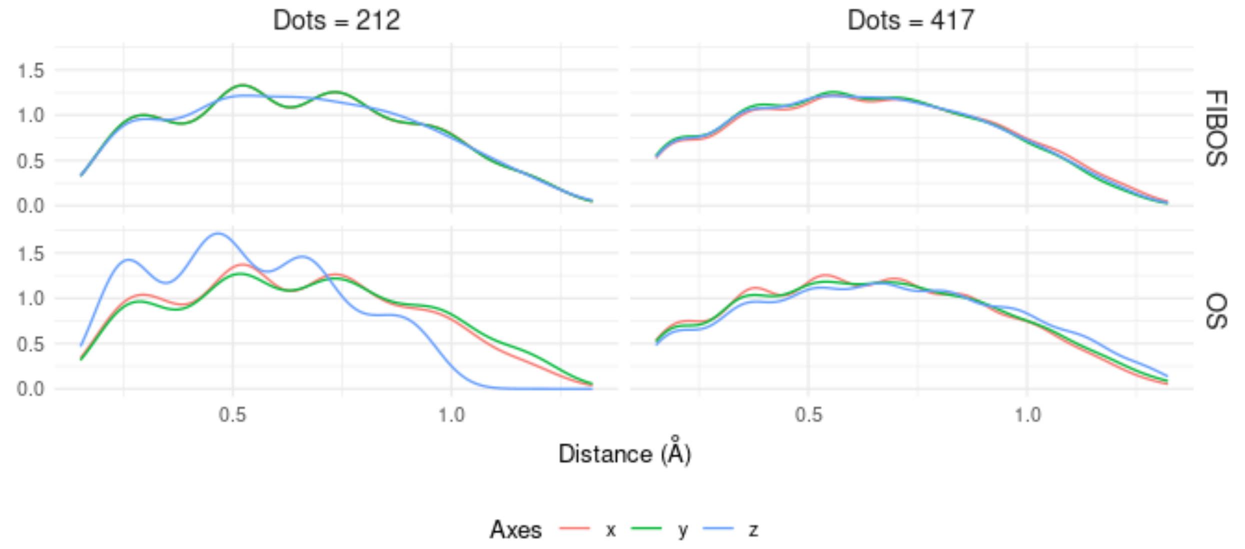


**Figure S2B.** Spherical cap distance distribution according to axes. Distance distribution of all dots from each other, but limited to spherical caps at 0.75 of the radius, on the x, y and z axes.

## 3. FIBOS COMPARISON IN FORTRAN, PYTHON AND R

The agreement of packing values calculated using these 3 programming languages is shown in Fig. S3.

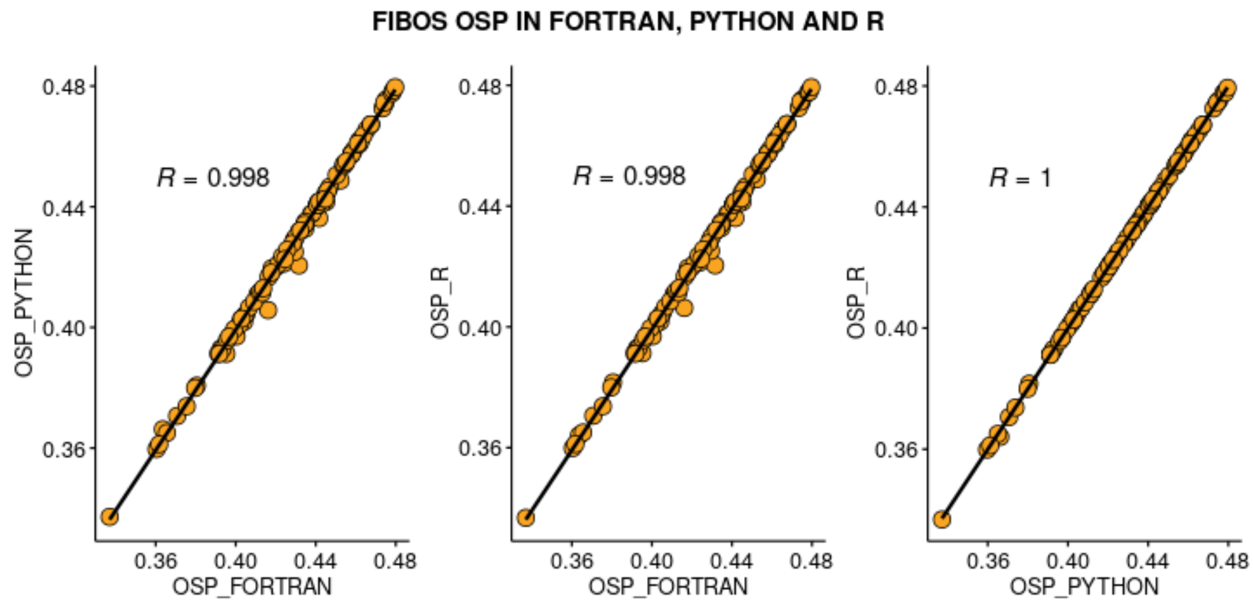


**Figure S3.** Linear regression indicating high correlations between OSP calculations between Fortran, R and Python, for validation dataset from Table S7.

# 4. METHODS

## 4.1. TERMINOLOGY

We are calling **EXP** the experimental models whose structures were resolved by X-ray diffraction, and **CSM** the computed structure models predicted by Alpha Fold (**AF**). We acknowledge the existence of other relevant structure predictors, such as RoseTTAFold (Krishna et al., 2024) and ESMFold (Lin et al., 2023). However, we focused on the AlphaFold models because they were more integrated with the RCSB PDB at the time this study was conducted. In future work, we plan to extend our analysis to include other computed structure models.

## 4.2 DATA SETS

The entire dataset construction process for the study case can be seen in Table S1. In summary, proteins were selected: with only one chain, with a size between 50 and 1200 residues, resolved by x-ray diffraction resolution less than or equal to 1.5, with sequence similarity cutoff up to 30%, without missing residues, which had associated CSM structures.

**TABLE S1 - PDB Dataset Creation Process for Study Case**

| STEP | SELECTION | CHAINS |
|---|---|---|
| 01 | Protein only; sequence length between 50 and 1200; polymer entity instance count equal to 1; assembly count equal to 1; method x-ray diffraction; resolution combined less than or equal to 1.5; similarity cutoff up to 30% | 2642 |
| 02 | Some PDB codes from step 01 were not available for download at the time. | 2613 |
| 03 | From step 02, filter the PDBs without missing residues in the middle of the chain | 1643 |
| 04 | From step 03, filter the PDBs that have Uniprot id associated | 1572 |
| 05 | From step 04, filter the PDBs with Alpha Fold (CSM) structure associated with an experimental structure (EXP) | 1455 |
| 06 | From step 05, filter the EXP PDBs without missing atoms and in which the sequence alignments between EXP and CSM do not present gaps in the middle of the chain due to lack of identity between residues (named extended dataset) | **746** |
| 07 | From step 05, filter the PDBs in which the difference in the number of residues between CSM and EXP does not exceed 5% of EXP (named strict dataset) | **261** |

A first dataset of 746 chains (164646 residues) was formed, called the extended data set (Table S8). It also included chains in which the number of residues in CSM is much higher than in EXP, such as 2CG7, which has 2477 residues in the AF model but only 90 in EXP. It is known that AF models are based on the entire canonical protein sequence, as defined by UniProt (Jumper et al. 2021).

To create a case of more directly comparable protein pairs, a second dataset was formed where the difference in the number of residues between CSM and EXP does not exceed 5% of EXP, producing the so-called strict dataset (Table S9), with 261 chains (70167 residues).

A third dataset was used for validation of packages in R and Python against Fortran, called the validation dataset. This dataset comprised 97 unique string PDB IDs from the original OS publication (Fleming and Richards 2000). This allowed for comparison of the Fortran calculations from the original publication with the new ones in R and Python. The validation dataset can be seen in Table S7.

### 4.3. OSP METRIC

The OSP metric is defined as (Fleming and Richards 2000):

$$OSP = \frac{\sum_{\text{atom}}^{\text{res}} \left[ OS_{\text{atom}} \cdot (1 - RL_{\text{atom}}) \right]}{MS_{\text{res}}} \qquad [1]$$

Where:

$OS_{\text{atom}}$ is the Occluded Surface (sum of areas under dots and normals). They are considered occluded because the normals indicate that they are very close to another sphere.

$RL_{\text{atom}}$ is the length of the extended normal divided by 2.8 Å (the diameter of a water molecule). Normals (and respective dots) larger than this value are eliminated, because since they did not find another sphere, their respective dots are considered as non-occluded areas.

$MS_{\text{res}}$ Is the total molecular surface of the residue (occluded and non-occluded areas)

How this equation works can be seen through a pictorial example in Fig. S4.

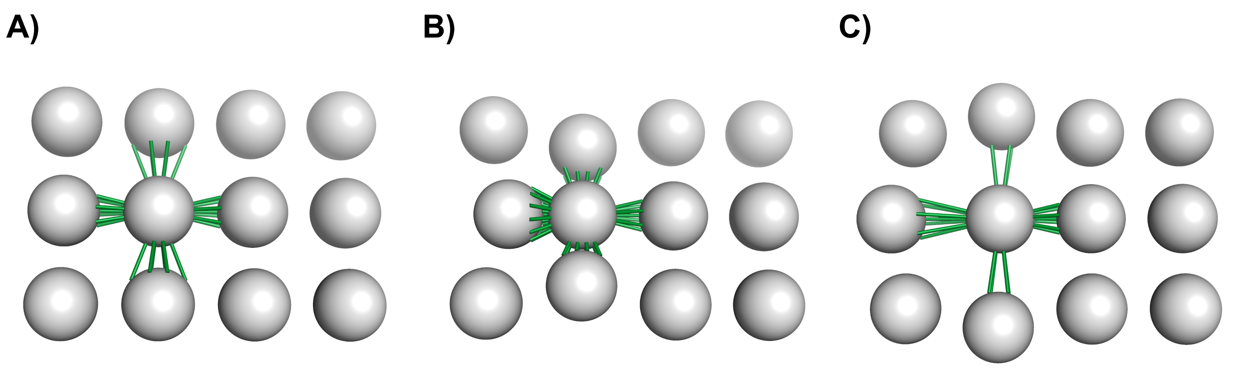


**Figure S4.** Illustrative example of how FIBOS uses dots and normals to measure packing. A) A lattice showing spheres in a more uniform spacing. From regularly distributed dots on the highlighted sphere, normals (green) are extended, only those that intersect the surface of a neighboring sphere are shown. B) If a given sphere has very close neighbors (more packed), a greater number of dots and short normals will be generated, making $OS_{\text{atom}}$ larger and $RL_{\text{atom}}$ smaller. The effect of this in equation [1] will be a larger OSP. C) If a given sphere has more distant neighbors (less packed), the opposite is true.

### 4.4 FIBOS CALCULATIONS

The `occluded_surface` and `osp` functions were used to calculate the OS at the atomic and residue levels per PDB ID, respectively, with default parameters. All scripts and dataset used for package validation and case study can be found at:
https://github.com/insilico-unifei/fibos-R-case-study-supp and
https://doi.org/10.5281/zenodo.15565375.

### 4.5 CLEANING OF PDBs

By default, the `occluded_surface` and `osp` functions 'clean' the input pdb file through an embedded shell script that checks and performs the following operations:

1. Filters the PDB file to keep only ATOM records and removes acetylated N-terminus (ACE);
2. Removes various types of hydrogens (H, 2H, 3H, D) and OXT atoms from the protein structure;
3. Deals with alternative conformations (occupancies) in the protein structure, keeping only the most frequent one.
4. Removes certain types of molecules that should be in HETATM records rather than ATOM records:
   - Water molecules (HOH)
   - Other molecules: PMS, FOR, ALK, ANI;
5. Performs some standardization of amino acid and atom names:
   - Converts HSD and HSE to HIS (different histidine states)

- Renames OT1 to O and OT2 to OXT (terminal oxygen atoms)
- Fixes isoleucine CD atom naming (CD to CD1);

6. Adds an END record at the end of the file

### 4.6 CSM AF ASSOCIATIONS TO EXP PDB

The RCSB RESTful API (1.47.5) was used to download metadata from PDB IDs, especially the Uniprot IDs and primary sequences (Rose et al. 2021). The Uniprot IDs served for composing the URLs needed to download the predictive models from the AF website (Varadi 2022). In this way, each CSM of AF was associated with the respective EXP PDB from the strict dataset.

### 4.7 INFLUENCE FUNCTIONS

Influence function (IF) is a classical tool in robust statistics that quantifies the local sensitivity of a statistical functional $T(F)$ to infinitesimal contamination at a point $x$ (Hampel 1974, Ichimura and Newey 2022). Formally, for a probability distribution $F$, the influence function is defined as:

$$\mathrm{IF}(x; T, F) = \lim_{\varepsilon \to 0} \frac{T\left((1-\varepsilon)F + \varepsilon\delta_x\right) - T(F)}{\varepsilon} \qquad [2]$$

where $\delta_x$ is the Dirac measure centered at $x$.

In our case, the functional of interest is the standard deviation, defined as:

$$T(F) = \sigma_F = \left[\int (x - \mu_F)^2 \, dF(x)\right]^{1/2}, \quad \text{with } \mu_F = \int x \, dF(x). \qquad [3]$$

Applying first-order functional differentiation, the influence function for the standard deviation is:

$$\boxed{\mathrm{IF}_\sigma(x; F) = \frac{(x - \mu_F)^2 - \sigma_F^2}{2\sigma_F}} \qquad [4]$$

A positive value for $IF$ indicates that $x$ increases dispersion (i.e., lies further from the mean), while a negative value indicates that $x$ contracts the spread of the distribution.

To compare the contribution to OSP standard deviations of matched $i$ residues across CSM and EXP models, we define the net IF influence as the difference between their respective influence functions:

$$\boxed{\mathrm{IF}_i^{\mathrm{net}} = \mathrm{IF}_\sigma(OSP.csm_i; F_{CSM}) - \mathrm{IF}_\sigma(OSP.exp_i; F_{EXP})} \qquad [5]$$

This net quantity reflects the relative influence of each matched residue pair on sd difference: $(\sigma_{CSM} - \sigma_{EXP})$. Thus, a positive $\mathrm{IF}_i^{\mathrm{net}}$ indicates that a matched residue $i$ had a more impactful OSP.csm on its sd than the OSP.exp on its sd. Negative, the inverse.

The IF values calculated for all 70167 residues of the strict dataset can be seen in the file FIBOS_by_residue-dataset-all-v1.csv at https://doi.org/10.5281/zenodo.15565375.

### 4.8. ALGORITHM FOR SELECTION OF INFLUENTIAL RESIDUES

The pseudocode of this algorithm can be seen in Fig. S5. Basically, it removes the most influential residuals calculated by Eq. [4] until there is no difference between the OSP standard deviations. If sd(CSM) > sd(EXP), remove in CSM; if sd(CSM) < sd(EXP), remove in EXP; if equal, do nothing.

```
Algorithm 1 - SetInfluentialResidues(data, netIF)
Require: data    ▷ list of records with fields residue_id, osp_exp, osp_csm ...
Require: netIF   ▷ mapping residue_id → net-IF score
Ensure: flagged  ▷ residue_ids whose removal flips the SD inequality
sd_exp ← sd(data.osp_exp); sd_csm ← sd(data.osp_csm)
if sd_csm >= sd_exp
    sd1 ← sd_csm; sd2 ← sd_exp; target ← "CSM"
else
    sd1 ← sd_exp; sd2 ← sd_csm; target ← "EXP"
sorted ← data.sort(key = netIF, descending)
flagged ← []
while sd1 > sd2
    res_id ← sorted.residue_id
    flagged.append(res_id)
    sorted.remove(res_id)
    sd1, sd2 ← recompute_sd(sorted, target)
return flagged
```

**Figure S5.** Pseudocode describing the selection of influential residues.

### 4.9. SOLVENT-ACCESSIBLE SURFACE AREA (ASA)

The solvent-accessible surface area (ASA) for each residue was computed using Biopython's implementation of the Shrake–Rupley algorithm (Shrake and Rupley, 1973), at the residue level and normalized by the maximum allowable ASA for that amino acid type (Tien et al., 2013). A residue was considered surface if ASA >= 0.25, following Schmidt et. al. 2017.

### 4.10. SECONDARY-STRUCTURE ELEMENTS (SSE)

To calculate the SSE percentages, the biotite Python package and its P-SEA algorithm (Labesse et al. 1997) were used, which classifies the residues into only 3 classes: helix, strand and coil.

## 5. RESULTS

### 5.1. PROTEIN PACKING AND IDENTIFICATION OF INFLUENTIAL RESIDUES

A comparison of the overall packing between experimental and computed structural models for the strict dataset are shown in Fig. S6. The individual protein mean packing is similar between experimental and computed models (Fig. S6A) but the standard deviations of individual residue packing values are greater for computed models than experimental models (Fig. S6B). The effects of algorithm 1 (Fig. S5) on the mean and standard deviation distributions of the recalculated OSPs can be seen in Fig. S6C and S6D; the whole protein packing values are only slightly changed and the residue level standard deviations are now essentially identical.

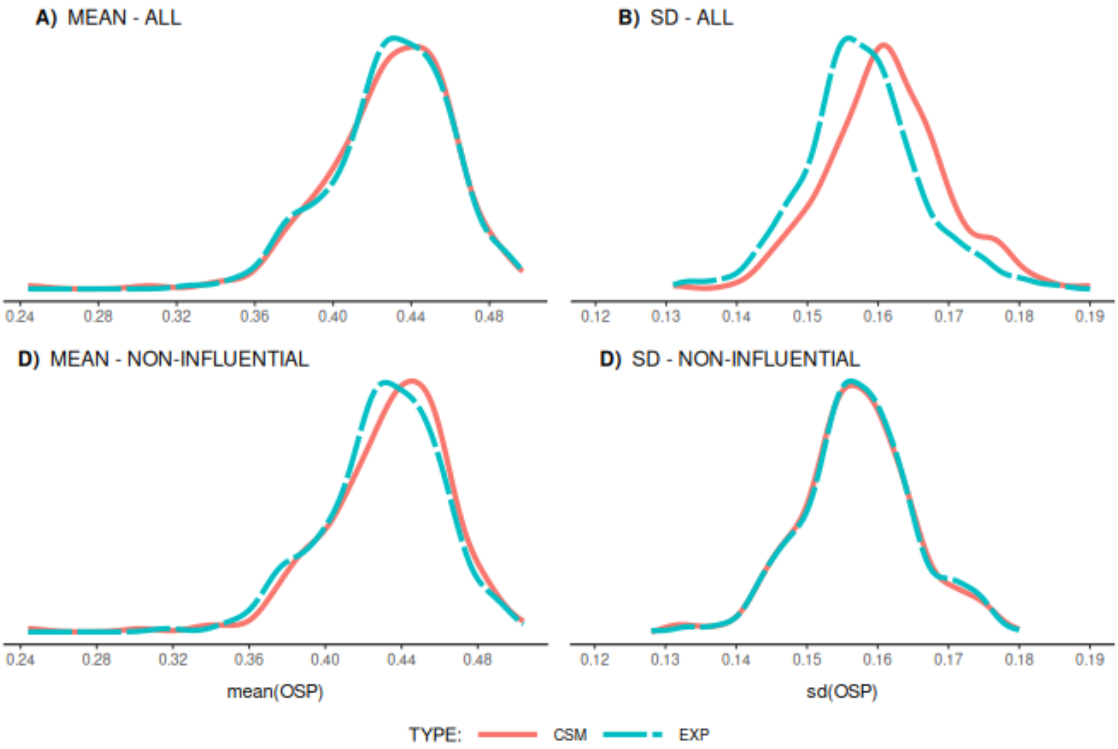


**Figure S6.** Protein packing density distributions involving the strict dataset of 261 chain structures, with experimental PDB (solid red lines) and predicted CSM (dashed blue lines). A) The density distributions of whole protein average residue OSP values. B) The density distributions of the standard deviation of residue OSP values. C) and D) The distribution of the means and standard deviations, respectively, filtered from their influential residuals, as described in Fig. S5.

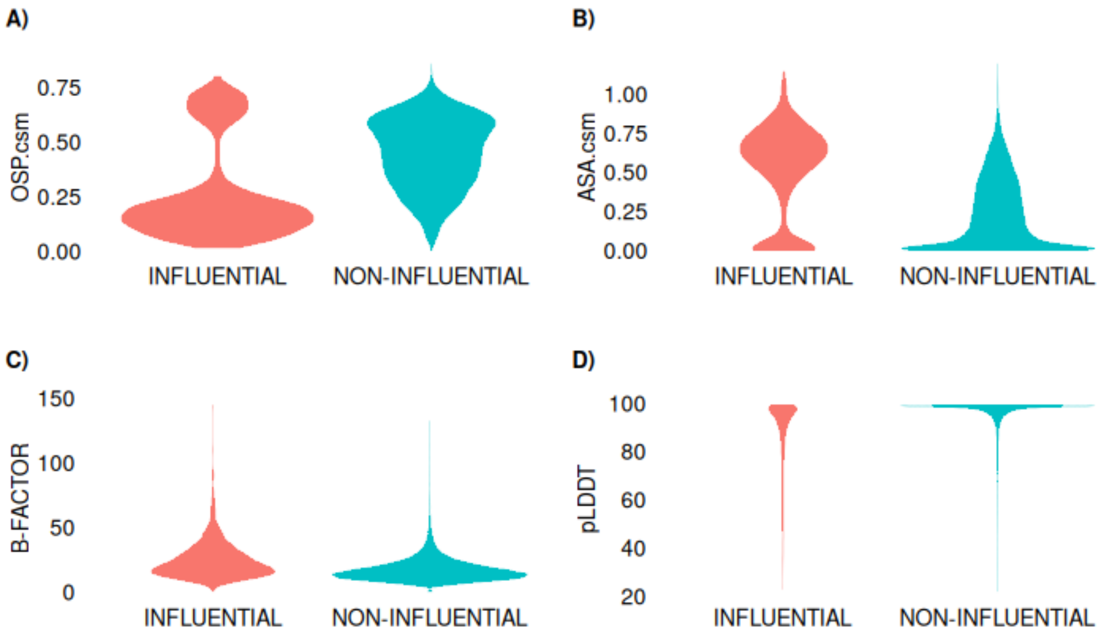


**Figure S7.** Comparison of some metrics between influential and non-influential residues: packing (OSP.csm), solvent accessible surface area (ASA.csm), B-factor and pLDDT. A) The most influential residues in CSM.sd are further away from the average (around 0.55), but in an asymmetrical way, with most of them being poorly packed. B) As expected, they are more exposed to the solvent. They also show a slight tendency for C) a higher B-Factor and D) a lower pLDDT.

Fig. S7 complements Fig. 1B of the main paper with the OSP, ASA, Factor-B and pLDDT metrics, in the comparison between influent and non-influent residues, reinforcing the pattern that non-influents tend to occur in residues exposed at the surface and in unstructured regions.

Eliminating residuals could have an impact on the recalculated mean and standard deviation. Statistically, for sd, although its Wilcoxon paired test shows a low p-value (<0.001), indicating a still consistent paired shift in the differences between sds, the magnitude of these effects by Cohen's d test is negligible (-0.017) (Table S5). Something similar happens for recalculated means: low p-value in Wilcoxon paired test (< 0.001) and negligible Cohen's d effect size (0.085) (Table S4). Therefore, the effect of the recalculation on the OSP distributions by Algorithm 1 was minimal and statistically acceptable.

**TABLE S2 - Statistical values for OSP mean (strict dataset)**

| | Wilcoxon Signed-Rank | Cohen's d effect size |
|---|---|---|
| data | `x = osp.csm.mean`<br>`y = osp.exp.mean` | `x = osp.csm.mean`<br>`y = osp.exp.mean` |
| R function | `wilcox.test(x, y, paired = TRUE, conf.int = TRUE)` | `cohen.d(x, y, paired = TRUE)` |
| Statistics | `(pseudo)median = -0.0000499`<br>`p-value = 0.958` | `d estimate: -0.0204 (negligible)` |

| | | |
|---|---|---|
| Confidence Interval by R function (0.95) | -0.00101 0.000997 | -0.0638 0.0230 |
| Confidence Interval by bootstrap (0.95) 1000 samples | -0.00213 0.000713 | - |

**TABLE S3 - Statistical values for OSP sd (strict dataset)**

| | Wilcoxon Signed-Rank | Cohen's d effect size |
|---|---|---|
| data | `x = osp.csm.sd`<br>`y = osp.exp.sd` | `x = osp.csm.sd`<br>`y = osp.exp.sd` |
| R function | `wilcox.test(x, y, paired = TRUE, conf.int = TRUE)` | `cohen.d(x, y, paired = TRUE)` |
| Statistics | (pseudo)median = 0.00395<br>p-value < 2.2e-16 | d estimate: 0.421 (near medium) |
| Confidence Interval by R function (0.95) | 0.00349 0.00446 | 0.346 0.496 |
| Confidence Interval by bootstrap (0.95) 1000 samples | 0.00291 0.00413 | - |

**TABLE S4 - Statistical values for OSP mean (strict dataset adjusted by algorithm 1)**

| | Wilcoxon Signed-Rank | Cohen's d effect size |
|---|---|---|
| data | `x = osp.csm.mean.adj`<br>`y = osp.exp.mean.adj` | `x = osp.csm.mean.adj`<br>`y = osp.exp.mean.adj` |
| R function | `wilcox.test(x, y, paired = TRUE, conf.int = TRUE)` | `cohen.d(x, y, paired = TRUE)` |
| Statistics | (pseudo)median = 0.00343<br>p-value = 3.206e-09 | d estimate: 0.0852 (negligible) |
| Confidence Interval by R function (0.95) | 0.00247 0.00450 | 0.0383 0.132 |
| Confidence Interval by bootstrap (0.95) 1000 samples | 0.00117 0.00406 | - |

**TABLE S5 - Statistical values for OSP sd (strict dataset adjusted by algorithm 1)**

| | Wilcoxon Signed-Rank | Cohen's d effect size |
|---|---|---|
| data | `x = osp.csm.sd.adj`<br>`y = osp.exp.sd.adj` | `x = osp.csm.sd.adj`<br>`y = osp.exp.sd.adj` |
| R function | `wilcox.test(x, y, paired = TRUE, conf.int = TRUE)` | `cohen.d(x, y, paired = TRUE)` |
| Statistics | (pseudo)median = -0.00103<br>p-value = 0.000180 | d estimate: -0.0169 (negligible) |

| | | |
|---|---|---|
| Confidence Interval by R function (0.95) | -0.00103 -0.000946 | -0.0266 -0.00726 |
| Confidence Interval by bootstrap (0.95) 1000 samples | -0.000230 -0.0000651 | - |

### 5.2. STRUCTURAL METRIC CORRELATIONS AT THE CHAIN LEVEL

We can highlight the following correlations between different metrics of the protein chains shown in Figs. S8A and S8B: Size_diff shows anticorrelation with pLDDT and OSP, indicating that CSM models expanded in number of residues (as defined by UniProt) in relation to EXP models have their prediction quality partially compromised, even when we consider only the residues aligned between CSM and EXP. OSP correlates positively with chain length and helix content, as already described in (Fleming and Richards 2000). A slight anticorrelation is observed between helix and osp.sd, indicating that the higher the helix content, the lower the OSP standard deviation. pLDDT_global correlates positively with higher packing (higher mean of OSP), but is indifferent to the variation of OSP.

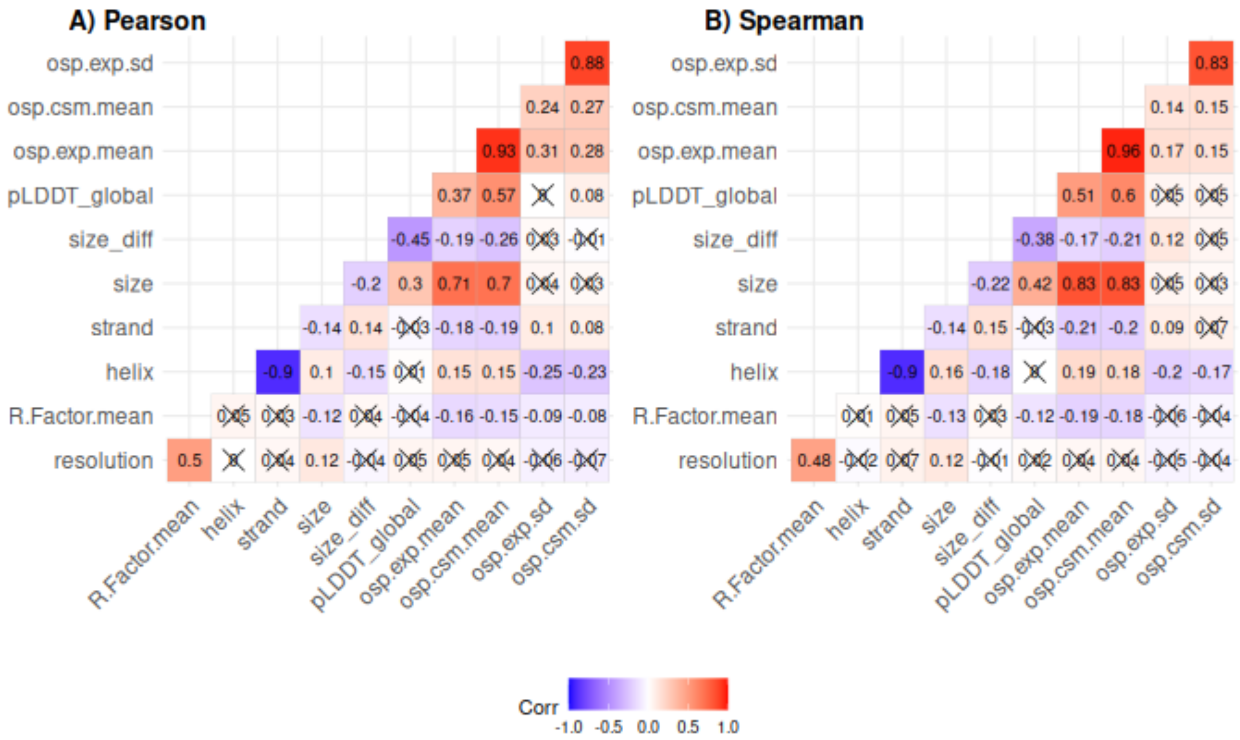


**Figure S8A.** Correlogram of the extended dataset involving 746 chains using A) Pearson and B) Spearman correlation. Diagonally cut squares represent statistical absence of correlations (at a confidence level of 0.95). The following structural metrics were considered: resolution; R.factor.mean, average of R_FACTOR and R_FACTOR_WORK; relative content of helix and strand secondary structure elements; size as the number of resídues; size_diff, the absolute difference in the number of residues between computer simulated models (CSM) and experimental (EXP); pLDDT_global, quality inference chain average for CSM models (higher is better, recalculated taking into account only the aligned CSM and EXP residues); osp.exp.mean, osp.csm.mean, osp.exp.sd, osp.csm.sd, average and standard deviation of OSP for EXP and CSM, respectively.

Even after performing a residue alignment and trimming the unmatched residues so that CSM and EXP have the same pairwise residues and are the same size, we found that these pairwise trimmed CSMs tend to produce a worse recalculated trimmed pLDDT_global. This can be seen in the correlogram in Fig. S8A, which shows for more appropriate comparison, both parametric (Person) and nonparametric (Spearman) correlation. The pLDDT_global (trimmed) presents an anticorrelation with this size_diff between CSM and EXP models around -0.40.

Because of this bias, a new filter was introduced, where the difference in the number of residues between CSM and EXP does not exceed 5% of EXP, producing the so-called strict dataset (Table S9), with 261 chains (70167 residues). It can be seen in Fig. S8B that in the strict dataset, the bias of pLDDT_global is eliminated, with it maintaining a noteworthy correlation only with OSP.means.

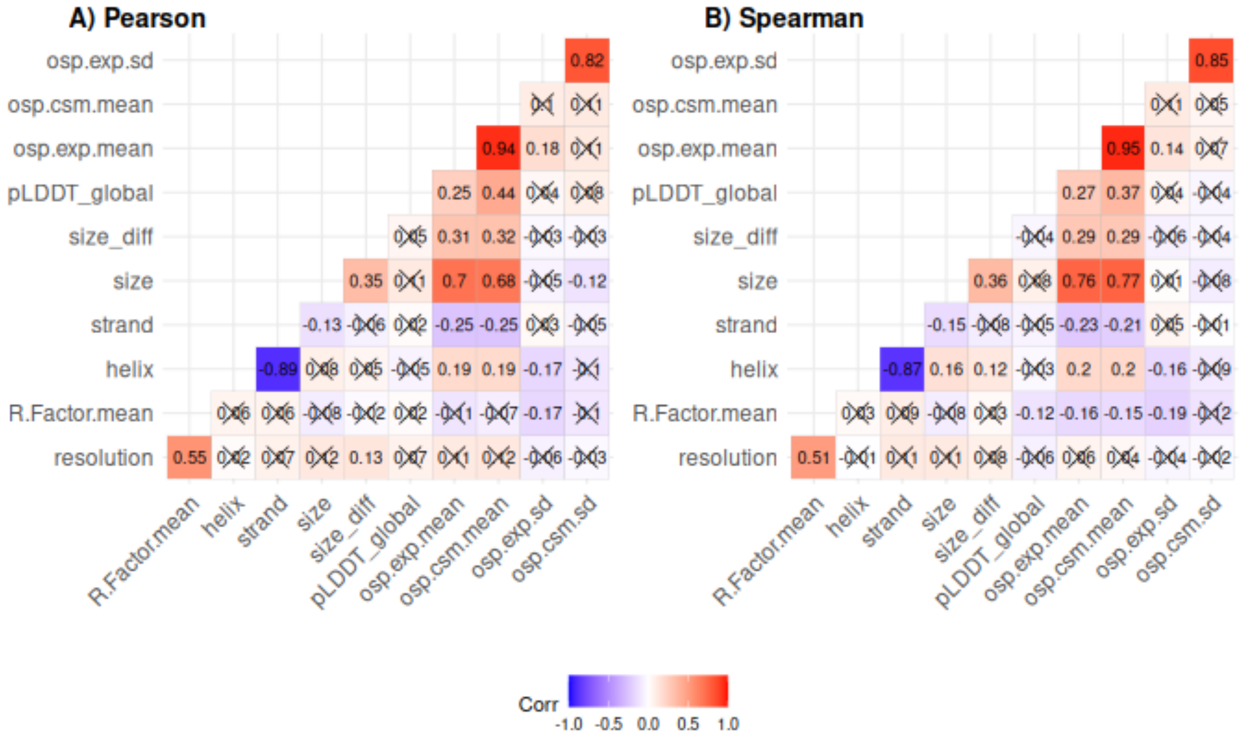


**Figure S8B.** Correlogram of the strict dataset involving 261 chains using A) Pearson and B) Spearman correlation. The attributes are the same as described in Fig. S8A.

### 5.3. EXAMPLE OF DIFFERENCE IN HYDROPHOBIC SIDE CHAIN PACKING

In the main text an example of a difference in the packing of a surface charged residue between EXP and CSM models is shown in Fig. 1C. A difference involving a hydrophobic residue is shown in Fig. S9 where there is a large difference in the packing of a leucine (LEU16) in a lipid binding protein. This difference is due to a neighboring phenylalanine (PHE105) existing in a different rotamer in the CSM model and blocking the ligand binding site. Since all CSM models are in apo form, this may be a consequence of conformational flexibility. But, If the CSM

structure were the only one available, this partially obstructed pocket could be a complication for docking algorithms.

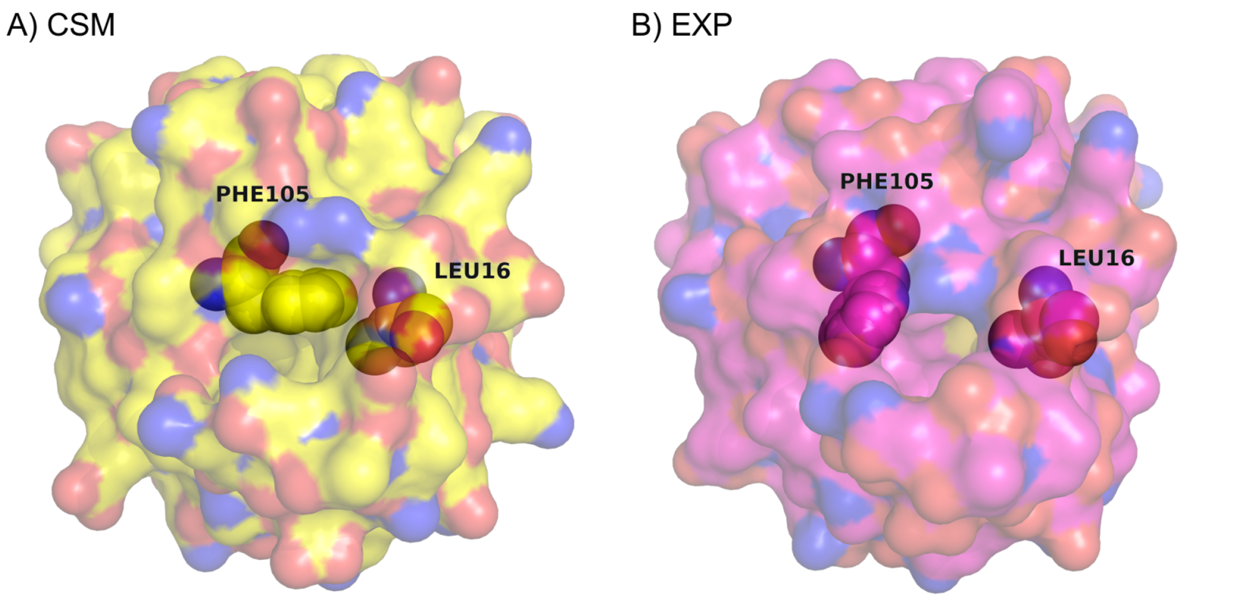


**Figure S9.** Example of influential residue LEU16 in the 1PZ4 core. In the CSM (unlike the EXP) the approach of the PHE105 to LEU16 partially obstructs the entrance to the ligand binding site.

### 5.4. PROTEIN CORE PACKING AND NUMBER OF INFLUENTIAL RESIDUES

It was recently reported that the average core packing fraction in proteins is 0.55 ± 0.01, using a Voronoi based algorithm on a dataset of ~5000 high-quality x-ray crystal structures (see Fig. 07 in Grigas et. al. 2025). The OSP packing of core-only residues in the strict dataset is shown in Fig. S10. The mean OSP packing value for core residues of the strict dataset is identical to the Voronoi based value previously reported by Grigas et al. 2025.

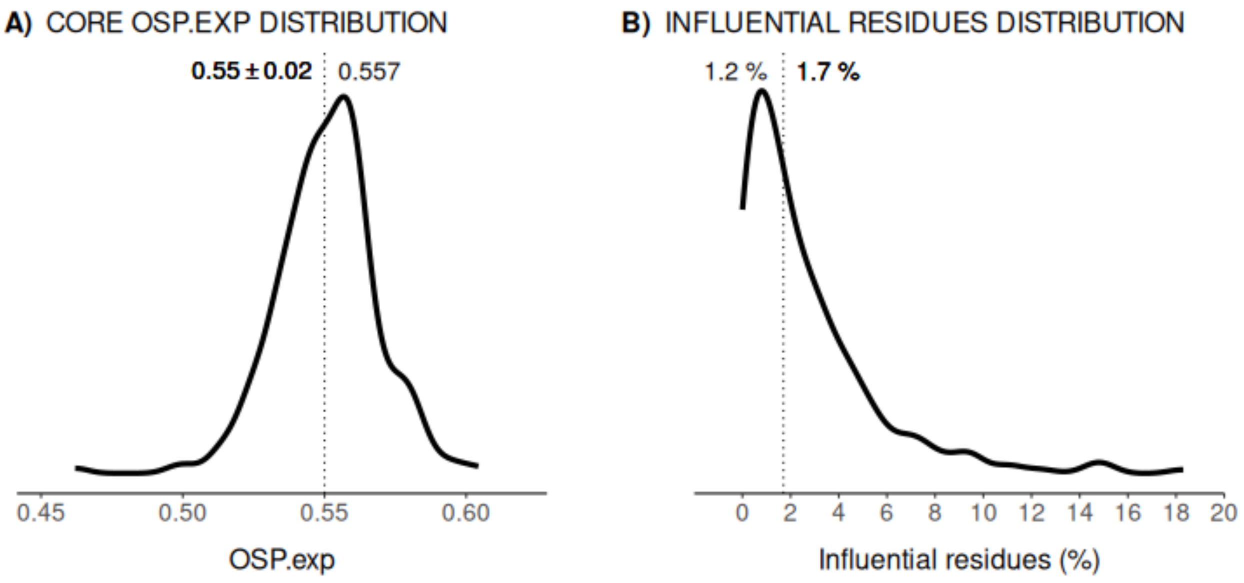

**Figure S10.** Density distributions using the strict dataset with 261 chains and 70167 residues. A) Packing density distribution of residues from the protein core for EXP models. Values in bold indicate mean and standard deviation. Value in plain text indicates the modal. B) Percentage of the number of influential residues by chain. Value in bold indicates the median. Value in plain text indicates the modal. In both plots the dotted line indicates the central tendency.

The number of residues that influence the larger standard deviations of CSM packing values is small for most models (median = 1.7%) although the distribution extends to 18% as seen in Fig. S10B.

## 6. FIBOS VS VORONOIA BENCHMARK

We selected Voronoia (Rother et al. 2009) for comparative benchmarking with FIBOS because, to the best of our knowledge, it is the only one that explicitly calculates atomic packing densities, offering a quantitative metric that is comparable to our occlusion-based approach.

However, it is important to note that methodologies that use Voronoi for density packing calculations (such as Voronoia) face challenges at surface points, because under these conditions, it can yield open cells, with infinite volumes (da Silveira et al. 2009). FIBOS does not use Voronoi and does not have this limitation.

We compared the runtime performance and memory (RAM) usage between FIBOS and Voronoia, in Python, all default parameters, for a sample of 100 PDB of EXP model, from the strict dataset, in quadruplicate. The result is shown in Table S6. A laptop with the following configuration was used: AMD RYZEN 5 5500U WITH RADEON GRAPHICS - 2.10GHz 16.0 GB RAM 238 GB HARD DRIVE WINDOWS 11 PRO 24H2. Voronoia Python script was taken from https://github.com/krother/Voronoia .

**TABLE S6 - Runtime and Memory (RAM) Usage for FIBOS and Voronoia**

| | FIBOS | VORONOIA |
|---|---|---|
| Total Runtime (hours) | 5.510 5.541 5.549 5.537 | 1.573 1.470 1.469 1.468 |
| mean ± sd | 5.534 ± 0.017 | 1.495 ± 0.052 |
| Max Memory (MB) | 179.2 174.3 177.1 174.6 | 20.7 20.7 21.7 21.2 |
| mean ± sd | 176.3 ± 2.3 | 21.1 ± 0.5 |

**TABLE S7:** Validation dataset.
FIBOS_SI_validation_dataset_2025

**TABLE S8:** Extended dataset.

FIBOS_SI_extended_dataset_2025

**TABLE S9:** Strict dataset.
FIBOS_SI_strict_dataset_2025

**TABLE S10:** Atomic radii adapted from https://pages.jh.edu/pfleming/sw/os.
FIBOS_SI_atom_radii_2025

## REFERENCES


da Silveira CH, Pires DE V, Minardi RC et al. Protein cutoff scanning: A comparative analysis of cutoff dependent and cutoff free methods for prospecting contacts in proteins. Proteins 2009;74:727–43.

Grigas AT, Liu Z, Logan JA, Shattuck MD, O'Hern CS. Protein Folding as a Jamming Transition. PRX Life [Internet]. 2025 Mar 27;3(1):013018.

Hampel FR. The Influence Curve and Its Role in Robust Estimation. Journal of the American Statistical Association. 1974;69(346):383–93. 1.

Ichimura H, Newey WK. The influence function of semiparametric estimators. Quantitative Economics. 2022;13(1):29–61.

Jumper J, Evans R, Pritzel A, Green T, Figurnov M, Ronneberger O, et al. Highly accurate protein structure prediction with AlphaFold. Nature. 2021;596(7873):583–9.

Krishna R et al. Generalized biomolecular modeling and design with RoseTTAFold All-Atom. Science (6693) 2024;384:p.eadl2528.

Labesse G, Colloc'h N, Pothier J, Rmornon J. P-SEA: a new efficient assignment of secondary structure from COL trace of proteins. CABIOS [Internet]. 1997;13(3):291–5.

Lin Z, Akin H, Rao R et al. Evolutionary-scale prediction of atomic-level protein structure with a language model. Science (6637) 2023;379:1123–30.

Rose Y, Duarte JM, Lowe R *et al.* RCSB Protein Data Bank: Architectural Advances Towards Integrated Searching and Efficient Access to Macromolecular Structure Data from the PDB Archive. *J Mol Biol* 2021;**433**:166704.

Rother K, Hildebrand PW, Goede A, Gruening B, Preissner R. Voronoia: Analyzing packing in protein structures. Nucleic Acids Research. 2009;37:2008–10.

Schmidt C, Macpherson JA, Lau AM, Tan KW, Fraternali F, Politis A. Surface Accessibility and Dynamics of Macromolecular Assemblies Probed by Covalent Labeling Mass Spectrometry and Integrative Modeling. Analytical Chemistry. 2017 Feb 7;89(3):1459–68.

Shrake A, Rupley JA. Environment and Exposure to Solvent of Protein Atoms. Lysozyme and Insulin. Vol. 79, J. Mol. Bid. 1973.

Tien MZ, Meyer AG, Sydykova DK, Spielman SJ, Wilke CO. Maximum allowed solvent accessibilites of residues in proteins. PLoS ONE. 2013 Nov 21;8(11).

Varadi M, Anyango S, Deshpande M *et al.* AlphaFold Protein Structure Database: Massively expanding the structural coverage of protein-sequence space with high-accuracy models. *Nucleic Acids Res* 2022;**50**:D439–44.
